# Zebrafish: A Suitable Tool for the Study of Cell Signaling in Bone

**DOI:** 10.3390/cells9081911

**Published:** 2020-08-17

**Authors:** Maria Teresa Valenti, Giulia Marchetto, Monica Mottes, Luca Dalle Carbonare

**Affiliations:** 1 Department of Medicine, University of Verona, Ple Scuro 10, 37100 Verona, Italy; giulia.marchetto@univr.it (G.M.); luca.dallecarbonare@univr.it (L.D.C.); 2 Department of Neurosciences, Biomedicine and Movement Sciences, University of Verona, 37100 Verona, Italy; monica.mottes@univr.it

**Keywords:** zebrafish, bone, cartilage, osteogenesis imperfecta

## Abstract

In recent decades, many studies using the zebrafish model organism have been performed. Zebrafish, providing genetic mutants and reporter transgenic lines, enable a great number of studies aiming at the investigation of signaling pathways involved in the osteoarticular system and at the identification of therapeutic tools for bone diseases. In this review, we will discuss studies which demonstrate that many signaling pathways are highly conserved between mammals and teleost and that genes involved in mammalian bone differentiation have orthologs in zebrafish. We will also discuss as human diseases, such as *osteogenesis imperfecta*, osteoarthritis, osteoporosis and Gaucher disease can be investigated in the zebrafish model.

## 1. Introduction

A peculiar feature of vertebrates is bone, a rigid but dynamic tissue giving support to the organism body structure from the inside. Vertebrate skeletons are remarkably similar. Centuries-old studies of fossils and bone morphology have demonstrated that skull, vertebrae, and appendicular skeleton originate from identical bones in fish, amphibians, reptiles, and mammals [1,2]. The predominant types of skeletal tissues, bone and cartilage, and the main categories of skeletal cells (chondroblasts, chondrocytes, osteoblasts, osteocytes, osteoclasts) are present in teleost as wells as in mammals [3]. Both humans and zebrafish develop endochondral and dermal bones as well as cartilage that persist in the adult [4]. Bone is formed after condensation of mesenchymal stem cells (MSCs), which can then undergo two main processes: dermal or intramembranous ossification, involving the direct differentiation of condensed MSCs into osteoblasts, and endochondral ossification, involving MSC differentiation into chondrocytes and formation of a cartilage template that will thereafter be replaced or surrounded by bone [5]. Although zebrafish develop a simple pattern of early larval cartilages and bones, this is just the early basic skeletal pattern that is highly conserved among all vertebrates [6]. However, we will report how similarities and differences are nano (molecules) - and macro (bone structure)-scale dependent, and how they reflect either aquatic or terrestrial living environments.

In recent years, genetic studies in humans, mice, and zebrafish have revealed that such similarities at the morphological and cellular level actually reflect homologies at the molecular level in the underlying mechanisms of skeletal morphogenesis [7]. In fact, complete zebrafish genome sequencing and analysis revealed that 71.4% of human genes have at least one ortholog in zebrafish [7], including bone-related genes. Zebrafish have a very small size, and females may deliver up to 100 eggs per week; 48 h after fertilization, all common vertebrate body features are visible. Bone formation steps can be followed in a more complete way than by using an in vitro system (e.g., iPSCs). Thanks to the transparency of embryos which develop outside the mother’s body, zebrafish allow performing of in vivo fluorescence imaging to monitor skeletogenesis and the crosstalk between bone cells, unlike other animal models, such as mouse and rat. Genome editing is also feasible in a rapid and precise manner, thus granting the production of models for various pathologies, including those related to bone [8]. Moreover, large clutches allow high-throughput drug or biomolecule screening in an easier manner compared to murine models: this possibility is very useful for studying pro-osteogenic or osteotoxic molecules [9].

In this review, we will discuss studies which have demonstrated that the major signaling pathways are highly conserved between mammals and teleost, and that the key regulators of skeletal development in mammals have orthologs in zebrafish, sharing significant sequence similarities and overlapping expression patterns [5,10,11]. Thus, zebrafish turns out to be an attractive and simple model to generate genetic mutant and fluorescent reporter transgenic lines. Such models allow numerous studies revealing genes and signaling pathways involved in cartilage/bone development and homeostasis as well as genetic mutations associated with bone diseases in humans, such as osteoporosis, osteoarthritis, osteogenesis imperfecta, and others.

## 2. Search Strategy

Studies related to cell signaling occurring in zebrafish bone formation were selected from public databases. We identified 429 full articles by consulting the following databases: PubMed, Web of Science, and Scopus. The search string used in all databases was zebrafish AND bone/OR mesenchymal cells/or osteoblasts/or skeletal. We then eliminated sorted duplicates and screened article abstracts for consistency with our review topic. A total of 62 articles related to the aim of the review were finally cited and reported in the References section.

## 3. Skeletal Tissue and Bone Formation

The skeletal cells and ossification process in zebrafish are similar to those of higher vertebrates. However, the zebrafish skeleton comprises a smaller number of cells.

The regulation of craniofacial development is conserved, and craniofacial elements present in zebrafish are similar to those of other vertebrates. It has been shown that craniofacial mesenchyme originates from the neural crest and paraxial mesoderm [12]. This dual origin significantly helps tracking in vivo tissues interactions as well as gene expression modulation in the formation of craniofacial structures. For example, it has been observed that the cranial neural crest cells originate from the neural ectoderm, in particular, from the dorsal and lateral region, and they are visible in embryos at 12-hour post-fertilization (hpf) [12]. Proteins of the Wnt family control, at least in part, the fate of neural crest cells during a pre-migration state [13].

Bone development in zebrafish reflects the mammalian process, since the same key regulator genes take part in osteoblast differentiation in both kinds of organisms. First of all, the mammalian *Runx2* gene is highly conserved and has two orthologs in zebrafish, *runx2a* and *runx2b* [10]. The presence of these two isoforms in zebrafish is the consequence of a specific genome-wide duplication in teleost [7] which show a pseudotetraploid genome. Duplicate genes have been retained through evolution when having divergence either in function or in the expression pattern [14]. Interestingly, while information is lacking on maternal *Runx2* role in mammalian oocytes, the fundamental role of maternal zebrafish *runx2b* is in ventral organizer formation during dorsoventral patterning of the embryo, and skeletal development has been shown to precede the zygotic expression of *runx2a* and *runx2b* [10]. *Runx2b* is under the negative control of *twist1a* and *twist1b* genes which are zebrafish orthologs of mammalian *Twist* coding for regulatory bHLH proteins [15,16].

Most zebrafish bones, with the exception of parasphenoid, show the sequential transcriptional hierarchy required for mammalian osteoblast differentiation. Three overlapping stages [11], each one characterized by a distinctive marker, can be recognized: an early stage with preosteoblast marker genes *runx2a* and *runx2b* expression; an intermediate stage with osterix gene expression; a mature stage with late differentiation marker expression: osteocalcin, osteonectin, collagens and other bone matrix genes [11,17]. The sequential activation of these genes coincides with the downregulation of early and intermediate markers and with the maintenance of downstream genes expression in mature osteoblasts.

In more detail, the initial maternal expression of *runx2b* is followed by the expression of embryonic *runx2a* [10]; both genes are expressed in cartilage and bone primordia. Thereafter, at 36 hpf osterix (*osx*) is expressed in the whole bony skeleton, together with *runx2a* and *runx2b*. *osx* is the zebrafish ortholog of another well conserved “bone gene”, called *Sp7* in mammals. Sp7/osterix is a zinc finger transcription factor expressed specifically by osteoblasts: it plays an essential role in the differentiation and maturation of osteoblasts and in osteocytes formation [18]. Finally, at the mature stage (by 120 hpf), expression of the two *runx2* and *osx* genes declines, while their target genes, coding for bone matrix proteins (among them collagens, osteonectin *(osn)* and osteocalcin [11]) are switched on. In the intermediate and late stages of osteoblasts differentiation, *Tcf7*, mediator of the WNT signaling pathway in zebrafish, also shows increased expression in developing dermal bones as well as *cvl2*, mediator of the BMP pathway, which is present also in all bone primordia [11]. Collagens are produced: collagen type I and collagen type II are the principal components of cartilage, minor collagens such as type X and XI help to stabilize the structure [19]. In zebrafish, *col10a1a* is an ortholog of mammalian *Col10a1* encoding the alpha chain of type X collagen, a short collagen chain expressed in hypertrophic chondrocytes during endochondral ossification; in zebrafish it is a direct downstream target of *osx* [20].

Hedgehog is another important signaling pathway involved in osteoblast differentiation and mineralization. Hedgehog is upregulated by *Gli2;* it stimulates osteoblast development and mineralization acting on autophagy. In fact, in zebrafish osteoblasts, the Hh signaling pathway is an upstream inhibitor of *atg5* gene, while the autophagic pathway inhibits osteoblast-related proteins Osterix, BMP2, and Col10a1 [21]. Furthermore, Shh (Sonic Hedgehog) stimulates osteoblastic differentiation and bone formation, upregulating *Sp7* [21] which, in turn, directly upregulates *col10a1* [20].

A sharp increase in the expression of genes coding for bone matrix proteins such as Sparc, Bglap, Spp1, and Col1a2 is appreciable up to 7 dpf (day post fertilization), followed by a strong decrease: this indicates that bone matrix is mostly formed at 7 dpf and that further ossification is mainly due to mineral deposition. Accordingly, the strong decrease in *osx* expression suggests osteoblast differentiation decreases [22].

The most important genes involved in bone formation are summarized in Table 1.

A well-known and highly appreciated feature of zebrafish is transparency at early stages. Along with easy genetic manipulation, it has prompted the generation of transgenic reporter lines that allow following the dynamics of cells and marker gene expression in vivo. In fact, for most of the main genes involved in bone formation, a transgenic reporter line exists, e.g., col10a1a:Citrine and Col2a1a:mCherry [23], sp7:GFP and sp7:mCherry, and BMP:GFP, to cite just some of them [24].

## 4. Genes Involved in Bone Formation and Zebrafish Models

*Runx2* gene has been investigated using a mouse-zebrafish hybrid panel (*runx2a*) and a 1-month old zebrafish cDNA library (*runx2b*) [10]. In wild type zebrafish embryos, it has been demonstrated that Runx2 is the dorsoventral patterning maternal factor [25]. Four *twist* genes (namely *twist 1a*, *twist1b*, *twist2*, *twist3*) have been detected by screening a 48 h embryonic expression library [15]. *twist1a*, *twist1b*, *twist2* are orthologs of the mammalian genes *twist1* and *twist2* [15] but not *twist3*. Importantly, a morpholino-mediated *twist* gene knockout in zebrafish has shown that both *twist 1a* and *twist 1b* but neither *twist 2* nor *twist 3* are crucial for correct skeletal development [16]. Niu et al. applied the genome-editing tool TALEN to create a zebrafish sp7 mutant line, providing a useful tool to investigate the *SP7*/*Osterix* gene [20]. The authors so demonstrated that *sp7/osterix* regulates *col10a1a* expression in zebrafish [20]. In addition, by applying the CRISPR/Cas9 genome-editing tool, two additional knockout *SP7* lines were obtained which contributed to demonstrate that SP7 regulates *dlx2b* and *bglap* genes expression and, consequently, tooth development and mineralization [26]. Importantly, by using transgenic line and chemical interference approaches, Felber et al. demonstrated that *Osterix* expression is regulated by FGF and Wnt/ß-Catenin signaling during the osteogenic differentiation [27]. Hedgehog (Hh) signaling pathway is involved in osteogenic differentiation as well. It has been observed, by using zebrafish larvae models and transgenic zebrafish line Tg (-2.2col10a1a:GFP), that Hh signaling regulates osteogenic differentiation by affecting the autophagic process [21]. Hh signaling is also involved in the caudal fin endoskeletal primordium patterning, as demonstrated by using the 2.2shh:gfp:ABC#15 transgenic line [28] as well as in perichondral osteoblast differentiation as demonstrated by using the patched mutants (ptc1^hu1602^ and ptc2^tj222^) [27]. As described above, the osteogenic differentiation process in zebrafish larvae can be split in three stages on the basis of gene expression patterns [11]. The same pattern occurs in mammals. Intriguingly, by performing morpholino-based knockdown with antisense oligonucleotides, Rotllant et al. demonstrated that *sparc* is expressed in a temporally and spatially specific manner, and that this gene is strongly expressed during the development of the inner ear and in pharynx cartilage [29]. As for collagen, the most abundant bone matrix protein in mammals, three genes, *col1a1a*, *col1a1b*, and *col1a2* coding for collagen type I α1, α3, and α2 chains, respectively, are present in zebrafish [30]. It has been demonstrated that these three collagen type I genes are expressed in a similar spatiotemporal manner during zebrafish development, suggesting their coregulation and interdependence [30].

It has been demonstrated in morpholino-mediated knockdown of zebrafish that the expression of *shox* gene can inhibit early osteogenic differentiation in order to maintain embryonic osteoprogenitor cells [31]. Sawada et al. demonstrated that *shox* expression is also involved in the calcification process during late osteogenic differentiation [32]. Another gene involved in zebrafish bone development is *akt2*, which plays an important role in the regulation of different processes in mammals. By using CRISPR/Cas9 technology, Zhang and coworkers generated *akt2*-KO zebrafish and demonstrated that *akt2* is necessary for fin ray development, but not for cartilage maturation [33]. Interestingly, the authors suggested *akt2* to play divergent roles in mice and zebrafish, respectively, despite its evolutionary conservation [33].

To investigate the role of BMPs (bone morphogenetic proteins), different studies have been performed in the zebrafish model. By extracting total RNA from zebrafish embryos and larvae, it has been demonstrated that *bmp3* is involved in the arch skeletal system development [34]. Studies of fin sections obtained after 48 to 72 h amputation of zebrafish treated with BMPR (BMP receptor) inhibitor led to the conclusion that BMP regulates negatively the Wnt/β-catenin pathway in regenerating osteoblasts [35]. The role of *bmp1a* in osteoblast differentiation has been demonstrated in zebrafish *frilly fins* mutant, which is characterized by an impaired ossification [33]. On the other hand, morpholino-based knockdown of *bmp2*, *bmp4*, and *bmp7* caused mild dorsalization [37]. Moreover, BMPs together with fibroblast growth factors (FGFs) and WNT as well as Hh proteins regulate head bone and cartilage formation in mammals [38]. In humans, FGFs act as autocrine, paracrine, or endocrine factors. Among them, FGF8 plays an important role in the pharyngeal arches and palate formation [38]. Gebuijs and coworkers found a size reduction in eight of the nine craniofacial cartilage structures in zebrafish mutant line fgf8^ati282^; they also observed that nine mineralized structures were totally o partially absent in homozygous or heterozygous larvae [38]. A double-mutant *nob/dgf* embryo, generated by the no bone (*nob*) mutant, characterized by the absence of mineralization, and the *dragonfish* (dgf) mutant, characterized by craniofacial and axial skeleton ectopic mineralization, encoding a loss-of-function allele of the ectonucleotide pyrophosphatase phosphodiesterase 1 (*enpp1*), clarified that Entpd5 plays an important role in mineralization and in phosphate homeostasis in zebrafish [39]. Among cartilage proteins, UCMA (or GRP), a γ-carboxyglutamate (Gla) protein, has been investigated in zebrafish by using morpholino-based knockdown [40]. The structure of the *ucma* gene is highly conserved in mammals and zebrafish: the latter have two genes, *ucmaa* and *ucmab*, both expressed in the skeletal tissue [40]. However, *ucmaa* knockdown impairs skeletal development and leads to a reduction of aggrecan and collagen II content in cartilage [40].

Fin amputation in zebrafish has allowed for in depth examination of the different mechanisms and cellular pathways. The study of Hippo pathway activators YAP and TAZ in fin during osteogenic regeneration demonstrated that YAP induces osteoblast differentiation through the modulation of Bmp signaling [41].

Due to the growing interest in zebrafish as a model for bone formation and homeostasis, the number of available transgenic lines and reporters has been increasing in recent times. In addition to the models reported above, other tools are currently used to investigate the role of bone related genes in zebrafish [24]. To name but a few examples, the transgenic lines Tg(5xBMPRE-Xla.Id3:GFP) for studying BMP responsive elements, TgBAC(col10a1a:Citrine) for studying collagen10a1a, Tg(Col2a1aBAC:mCherry) for studying collagen2a1a, TgBAC(ctsk:Citrine) for studying cathepsin k, an enzyme involved in osteoclast activation, Tg(rankl:HSE:CFP) for studying rankl, a cytokine involved in osteoblast-osteoclast crosstalk.

## 5. Techniques for the Study of Bone Formation and Homeostasis in Zebrafish

Specific technical approaches have been developed to investigate osteogenesis in the zebrafish model. Larvae are very useful for studying bone formation. As zebrafish larvae are transparent and develop very quickly, they can be dynamically observed in vivo by using fluorescent transgenic lines to investigate early steps of skeletal formation.

Osteoblasts and osteoclasts must be monitored in order to evaluate bone homeostasis. Both these cell types can be labeled and observed in vivo by using osteoblast reporters (i.e., *sp7*) and osteoclast reporters (i.e., *ctsk*). Furthermore, simple staining techniques, e.g., Alizarin Red (AR) or Calcein staining which mark calcification areas, or Alcian blue that stains cartilage, represent additional investigative tools [24]. Fin regeneration assays, performed by using excised fins, associated with in vivo staining methods, such as AR (red fluorescence) and Calcein staining (green fluorescence), are very useful to study either dedifferentiation or differentiation processes as well as to assay bioactive molecules [24]. If, on the one hand, studies related to bone cells differentiation can be easily performed in zebrafish larvae by using transgenic reporters lines, then investigations of skeletal disorders associated to human adult phenotypes, on the other hand, need to be performed on the adult zebrafish skeleton. Therefore, histological staining, which allows visualization of osteoblasts, osteoclasts, and osteocytes, is often used. Frequently used techniques are in situ hybridization to detect osteoblast marker mRNAs (e.g., *osterix, bglap* mRNAs), or osteoclast markers (e.g., *ctsk* mRNA) as well as assays for the detection of alkaline phosphatase (ALP) activity in osteoblasts or of tartrate-resistant acid phosphatase (TRAP) activity in osteoclasts [42,43]. Osteocytes can be observed in hematoxylin–eosin-stained sections of mineralized bone matrix [44].

X-ray imaging techniques, such as radiographs, micro-computed tomography (μCT) or synchrotron equipped μCT technologies (SR-μCT) can be used to nondestructively investigate the fish. Moreover, X-ray imaging preserves samples for additional investigations (e.g., histology).

## 6. Bone Remodeling

Bone remodeling is a delicate and complex process for tissue homeostasis maintenance. Osteoblasts (bone forming cells) and osteoclasts (bone reabsorbing cells) are guided by mechanosensitive osteocytes embedded in the mineralized matrix [45].

For a long time, mice and rats have been the elective animal models for studying bone remodeling. In the last years, teleost ray-finned fish species have been used as well, thanks to the highly conserved skeleton [46]. Zebrafish skeleton shows similarities and differences when compared to the mammalian. Differences can be drawn on the basis of functional requirements, since terrestrial mammals are characterized by quadru- and bipedalism features. In addition, gravitational forces exerted on mammals and on fish are quite different, consequently affecting the remodeling processes in different ways. In fact, the zebrafish skeleton, being in an aquatic environment, is subjected to a lower mechanical load, although swimming can affect skeletogenesis in larvae [47] and bone remodeling in adults [45]. The adaptation to mechanical loading consequent to bi-quadrupedalism of terrestrial mammals is reflected by a different mineral density [48].

Skeleton hierarchical structure is the same in zebrafish and mammals. Therefore, the similarities and differences between zebrafish and mammalian skeleton are nano (molecules) - and macro (bone structure)-scale dependent [8]. At nanoscale levels, bone is constituted by a mineralized organic matrix which is primarily composed of collagen type I, while in humans, collagen type I is a heterotrimeric molecule [α1(I)_2_ α2(I)], in zebrafish, collagen type I is composed of three different α chains, α1(I), α2(I), and α3(I) [30]. At macroscale levels, it is important to report that endochondral bones prevail in human skeleton, while in zebrafish, cartilaginous bones are limited to the craniofacial skeleton. In addition, cortical bone is not present in zebrafish and hematopoietic bone marrow is not bone-encapsulated in zebrafish [8].

During the transparent early life stages, the zebrafish allow for analysis of the developing osseous structures. In addition, teeth replacement throughout life and the regeneration of skeletal structures, such as scales and fin rays occurring in zebrafish, make it a precious model for evaluating the remodeling process [45]. A major player in this process is RANKL, which is produced by osteoblasts, and induces osteoclast maturation and activation by binding their surface receptor RANK [49]. Osteoblasts produce also osteoprotegerin, OPG, which in turn can act as a RANKL decoy receptor and, competing with RANK, prevent bone resorption [50]. OPG protective action is exerted either by blocking osteoclasts’ maturation or by reducing their survival [50]. Interestingly, by studying the remodeling process in zebrafish, it has been demonstrated that osteoclasts control bone metabolism through the expression of semaphorin 4D, another player involved in this complex cellular crosstalk, which is induced by static acceleration [51]. Osteoclastic semaphorin 4D suppresses bone formation in humans too [52].

Zebrafish may represent a useful model to study mechanical properties of bone cells and skeletal and cartilage tissues. As reported above zebrafish, compared to mammals, are exposed to a lower mechanical loading but a higher mechanical stimulation, since they grow and live in the presence of a constant water flow. It is possible to mimic exercise and mechanical stimulation by modulating the water flow in proper machineries in order to monitor bone adaptation in response to musculoskeletal exercise, i.e., higher bone formation rate and higher bone mineralization [45]. Furthermore, zebrafish allow also to study microgravity effects on bone physiology by means of clinorotation experiments, which simulate the weightlessness condition experienced by astronauts in the space, but also the effects of prolonged bed immobilization. These experiments demonstrate a quite fast and significant decrease in bone formation in zebrafish larvae affecting FOS-JUN transcription factor complex and cAMP-responsive CREB1/CREM pathway [22].

## 7. Bone Regeneration

In mammals, the capacity to repair bone defects is limited because of restricted osteoblast potentiality, particularly reduced in the elderly and in pathological conditions [53,54,55] is and completely absent in case of large bone lesions or appendage amputation. By contrast, zebrafish is able to regenerate a variety of tissues, including bone, and whole body parts such as fins―following amputation―by completely and repeatedly restoring size, shape, and tissue patterning [56]. After fin amputation, mature osteoblasts dedifferentiate into preosteoblasts by losing the mature osteoblast marker bglap/osteocalcin and then migrate toward the amputation/regeneration site, where they re-differentiate, maintaining their commitment to the osteoblast fate. The transcriptional pattern hierarchy seen before is conserved, with the expression of *runx2* strongly activated after 2 days, *osterix* at day 3, and subsequent induction of *osteocalcin/bglap a*t days 5–6 followed by the downregulation of early and intermediate markers [17]. Dedifferentiation of osteoblasts is inhibited by NF-kB signaling. In differentiated *bglap*-expressing osteoblasts NF-kB is active, but it is inhibited following fin amputation, along with *bglap* expression; thereafter, dedifferentiated osteoblasts start to express preosteoblast markers [57].

At the fin amputation injury site, however, zebrafish present also regenerative growth with de novo osteoblasts differentiation from osteoblast progenitor cells (OPC). OPCs derive from embryonic somites and express matrix metalloproteinase 9 (mmp9); they are located in bone-forming tissue niches and migrate to the regeneration site, starting to express differentiation markers such as *osx*/*Sp7.* [58].

While the molecular mechanisms underlying fin regeneration (either from mature osteoblasts differentiation or from de novo osteoblasts production) mainly reflect those of development, substantial differences are to be found in adult zebrafish jaw regeneration. Zebrafish jaw is normally formed by intramembranous ossification; however, following resection, it is rebuilt through a cartilage intermediate. The periosteum, which produces osteoblasts under adult homeostasis conditions, starts producing chondrocytes, which express high levels of osteoblast-associated genes in contrast to what happens during development. Furthermore, Indian hedgehog a (Ihha), which is dispensable during customary chondrogenic development, turns out to be essential for inducing chondrogenic differentiation in the periosteum after injury [59].

The Hippo pathway is involved both in bone formation and repair by acting on commitment and differentiation of the osteoblast lineage [60]. It has been suggested that the Hippo pathway transcriptional co-activators YAP and TAZ maintain progenitor cells pluripotency and prevent osteoblast maturation [61]. On the contrary, other studies have suggested that the Hippo pathway stimulates osteoprogenitor cells differentiation and bone formation both in vitro and in animal models [62,63]. However, the different cell signaling effects promoted by Hippo pathway might vary in a context-dependent manner [64]. In this regard, a zebrafish regeneration model has been used to clarify Hippo pathway’s role in the differentiation and maturation of osteoblast lineage [41]. The authors, by using the zebrafish caudal fin regeneration model, addressed a new mechanism promoted by Hippo–Yap pathway, suggesting Yap involvement in the signaling cascade regulating osteoprogenitor maintenance and subsequent cell differentiation. In particular, they demonstrated that osteoprogenitors rack up, and their differentiation is prevented when Yap is inhibited. On the contrary, Yap expression induces Bmp2a production in mesenchymal cells located in proximal regions, thus enabling BMP signaling activation in adjacent osteoblasts, promoting their maturation [41].

## 8. Zebrafish and Human Bone Diseases

Zebrafish can be studied in the adult stage: this is an additional and interesting advantage since most skeletal disorders in humans appear in adults rather than in embryos. Furthermore, the zebrafish model often allows the study of homozygous mutants for genes, the mammalian orthologs of which are lethal: in this way, it is possible to investigate gene functions that cannot be addressed otherwise.

This is the case of *sp7 −/−* zebrafish mutants which have the deletion of all three zinc-finger domains of Sp7 and are a model of a severe recessive form of human osteogenesis imperfecta (type XII, OMIM# 613849) which, instead, is characterized by the deletion of only the last zinc-finger domain. Zebrafish *sp7 −/−* mutant can survive up to one year (adulthood) with severe bone and cartilage defects, while the corresponding murine model dies within 15 minutes from birth as a result of respiratory failure due to the lack of thoracic bones. The zebrafish model also allowed for the discovery that in adults, Sp7 directly regulates *col10a1* expression by binding to the two Sp1 sites in its promoter region. Sp7 regulation of *COL10A1* gene had never been observed in mammals [20].

Another recessive form of OI (OI type XIII OMIM#614856) I is due to mutations in *BMP1* gene, coding for a protease which is essential for the formation of mature collagen.

A zebrafish model for this condition is the *frilly fins* mutant [36] which shows delayed ossification and abnormalities of the vertebrae, revealing that BMP1 is required to produce mature osteoid [46].

Zebrafish *Chihuahua* (chi/+) is a popular model for dominant OI. It carries a heterozygous glycine substitution in *col1a1,* orthologous to the human *COL1A1* gene, encoding the α1(I) chain. Glycine substitutions in either *COL1A1* or *COL1A2* genes are involved in the majority of dominant OI cases in humans [65]. Phenotypic and molecular analysis, radiological screening of bone abnormalities [65] and micro-computed tomography (micro-CT) of skeletal morphology and bone microstructures [66] have demonstrated the suitability of zebrafish *chihuahua* as an animal model for human dominant OI.

Interestingly, a syndromic form of OI (Cole-Carpenter syndrome 2, OMIM#616294) characterized by disturbed ossification and craniofacial malformations, has been ascribed, upon whole exome sequencing, to compound heterozygous mutations in *SEC24D* gene [67]. *SEC24D* encodes a member of the COPII machinery, which is involved in procollagen I export from the endoplasmic reticulum. Once again, the corresponding zebrafish Sec24d mutant (*bulldog*) has been very useful since its cellular and morphological features mimic the human phenotype.

As for other connective tissue disorders, it is worth mentioning *col11a2 −/−* zebrafish mutants, which are vital until adulthood (contrarily to KO mice). They may represent a model for Stickler syndrome (OMIM#604841) and early onset osteoarthritis. This zebrafish model also allows to investigate the ancillary role of collagen type XI: the reciprocal interaction is important in order to maintain the diameter and spacing of type II collagen fibrils. In fact, even though type II collagen expression is not affected by *Col11a2* knockdown, its organization is largely impaired within 7dpf. The mutant phenotype shows alterations of jaw and joint morphology during development, leading to premature onset of OA in the adult, with abnormal collagen organization, degeneration of joint cartilage, and loss of joint space [19].

Studies have revealed that genes such as *Mcf2l*, *Pthrp*, *Col2a1*, and *Col9a2*, associated with osteoarthritis, are dynamically expressed during zebrafish development. The expression pattern of *Mcf2l*, a guanine nucleotide exchange factor, has been identified for the first time in zebrafish [23].

Other rare diseases inducing osteosclerosis have been studied using a zebrafish model. Gaucher disease, for example, has been studied with a transient *Gba1* knockout embryo. The aim was to clarify the molecular basis of the disease, considering that an impairment of osteoblastic activity associated to enhanced macrophage-dependent bone resorption might be a possible pathogenetic mechanism [68,69].

Among human skeletal disorders with the highest impact on life quality of affected people and healthcare costs, osteoporosis (OP) holds a prominent place. This degenerative bone disease is characterized by reduced bone mineral density (BMD) and bone mass (BM) with brittle bones prone to fracture; zebrafish turns out to be a very useful model for studying this [24]. A study on zebrafish helped to unravel the role of Smad9 as a downstream inhibitor of the BMP signaling pathway and reducer of osteoblast activity, providing evidence that Smad9 can be used as anabolic target for the treatment of osteoporosis. A loss-of-function *SMAD9* mutation reduces BMP inhibition which, in turn, allows enhanced bone formation through the positive regulation of RUNX2 by BMP2 [70]. OP-like symptoms can also be induced in wild type or bone transgenic zebrafish using the glucocorticoid dexamethasone; this model has been extensively used in recent years to test potential pharmacological treatments, which in zebrafish, can be easily performed on a large scale. Resveratrol [71], flavonoids [72], alendronate, and several kinase inhibitors acting on bone mineralization [73] have been tested among other potential treatments.

A degenerative skeletal disease such as osteoarthritis has been investigated using for example *Mcf2l*, *Gdf5*, *Col9a2*, *col11a2* zebrafish mutants [74]. On the other hand, *ptk7* mutants have been studied as models for idiopathic scoliosis [75], confirming the utility of zebrafish models in functional studies of genes involved in skeletal diseases.

## 9. Conclusions

**Figure 1:**
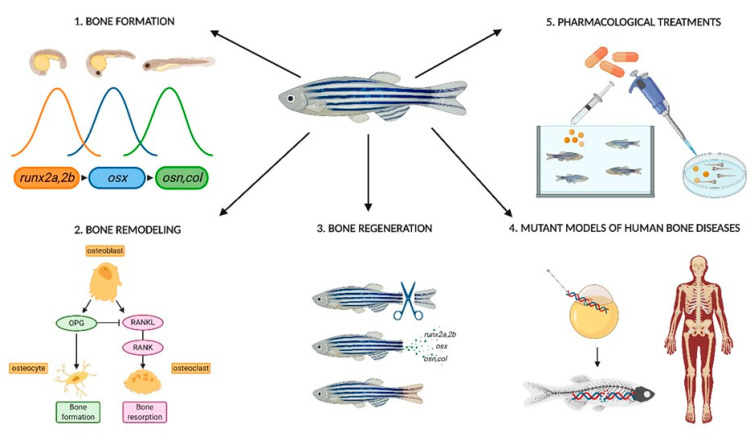
Zebrafish is a suitable tool for the study of cell signaling in bone, starting from the bone formation mechanism which recapitulates mammals molecular pattern involving orthologous genes runx2a/runx2b, osterix (osx) and downstream genes coding for bone matrix proteins (e.g., osteonectin (osn), osteopontin and collagens), (**1**). Mammalian bone homeostasis and remodeling processes are recapitulated in zebrafish, since they involve the same molecular pathway: high levels of RANKL produced by osteoblast (ob) bind to RANK receptor on preosteoclasts inducing osteoclast (oc) maturation and consequent bone reabsorption. Osteoprotegerin (OPG) inhibits RANKL–RANK binding by acting as decoy receptor allowing osteocyte maturation and thus bone formation (**2**). Notably, a very peculiar characteristic in zebrafish is the ability to regenerate entirely functional appendages after injury, such as the caudal fin after cut (**3**). Regeneration is accomplished by dedifferentiating mature osteoblasts into precursors and resetting the bone formation signaling pattern made of sequential activation of *runx2, osx*, and genes coding for bone matrix proteins. Zebrafish also grants feasible genetic manipulation and investigations in the adult stage at bearable costs. This is a valuable advantage since some human degenerative skeletal disorders are phenotypically evident in adults. Zebrafish mutants for human bone disorders can be generated at ease and furthermore studied also in the homozygous condition, which may result lethal in the mammalian orthologs (**4**). In this way, it is possible to investigate gene functions that cannot be addressed otherwise and also to test possible pharmacological treatments both in the adult and larval stages in a high number of individuals (**5**). Created with BioRender.com.

In conclusion, zebrafish is an excellent model organism for the study of the osteoarticular system (Figure 1), both at the embryonic and at the adult state, for distinct and complementary reasons that we have discussed in this review.

## Figures and Tables

**Table 1:** Genes involved in bone formation in zebrafish.

Gene/Protein	Role	Reference
*Runx2*	Transcription factor that triggers mesenchymal stem cell commitment towards osteogenic differentiation	[10,25]
*Twist*	Regulation of skeletal development and dorsoventral patterning	[15,16]
*Sp7/Osterix*	Transcription factor, Runx2 downstream regulator of osteogenic differentiation	[18,20,26]
*Bglap/Osteocalcin*	Bone mineralization-related gene	[25]
*Gli2*	Gene involved in hedgehog (Hh) signaling regulating osteoblast differentiation	[21,27,28]
*Sparc/Osteonectin*	Bone mineralization-related gene	[11,29]
*Col1a1a*, *Col1a1b*, *Col1a2/Collagen type I*	Bone ECM most abundant protein	[30]
*shox/SHOX* (short-stature homeobox-containing gene)	Regulator of cell proliferation and bone differentiation	[31,32]
*akt2*	Promotes bone development	[33]
*BMPs* (bone morphogenetic proteins)	Group of growth factors promoting the formation of bone and cartilage	[34,35,36,37]
*Fgf8a /Fibroblast growth factor*	Regulates skeletal genes expression	[38]
*Entpd5/* *Ectonucleoside triphosphate diphosphohydrolase 5*	Regulates phosphate homeostasis for skeletal mineralization	[39]
*Grp-2/ UCMA*	Coding for “unique cartilage matrix associated protein” required for skeletal development	[40]
*YAP/TAZ* genes	Coding for Yes-associated protein (YAP) and transcriptional coactivator with PDZ-binding motif (TAZ)- Hippo pathway transcriptional co-activators involved in osteoblast differentiation	[41]

## References

[1] De Beer G.R. (1937). The Development on the Vertebrate Skull.

[2] Goodrich E.S. (1988). Studies on the Structure and Development of Vertebrates. J. Hist. Biol..

[3] Apschner A., Schulte-Merker S., Witten P.E. (2011). Not all bones are created equal—Using zebrafish and other teleost species in osteogenesis research. Methods Cell Biol..

[4] Hall B., Hanken J. (1985). Foreward to GR de Beer’s, the Development of the Vertebrate Skull.

[5] Spoorendonk K.M., Hammond C.L., Huitema L.F.A., Vanoevelen J., Schulte-Merker S. (2010). Zebrafish as a Unique Model System in Bone Research: The Power of Genetics Andin Vivoimaging. J. Appl. Ichthyol..

[6] Javidan Y., Schilling T.F. (2004). Development of Cartilage and Bone. Methods Cell Biol..

[7] Howe K., Clark M.D., Torroja C.F., Torrance J., Berthelot C., Muffato M., Collins J.E., Humphray S., McLaren K., Matthews L. (2013). The zebrafish reference genome sequence and its relationship to the human genome. Nature.

[8] Kwon R.Y., Watson C.J., Karasik D. (2019). Using Zebrafish to Study Skeletal Genomics. Bone.

[9] Laizé V., Gavaia P.J., Cancela M.L. (2014). Fish: A Suitable System to Model Human Bone Disorders and Discover Drugs with Osteogenic or Osteotoxic Activities. Drug Discov. Today.

[10] Flores M.V., Tsang V.W.K., Hu W., Zylińska M., Postlethwait J., Crosier P., Crosier K., Fisher S. (2004). Duplicate Zebrafish Runx2 Orthologues Are Expressed in Developing Skeletal Elements. Gene Expr. Patterns.

[11] Li N., Felber K., Elks P., Croucher P., Roehl H.H., Elks P.M. (2009). Tracking Gene Expression during Zebrafish Osteoblast Differentiation. Dev. Dyn..

[12] Schilling T.F., Kimmel C.B. (1994). Segment and Cell Type Lineage Restrictions During Pharyngeal Arch Development in the Zebrafish Embryo. Development.

[13] Dorsky R.I., Moon R.T., Raible D.W. (1998). Control of Neural Crest Cell Fate by the Wnt Signalling Pathway. Nature.

[14] Prince V.E., Pickett F.B. (2002). Splitting Pairs: The Diverging Fates of Duplicated Genes. Nat. Rev. Genet..

[15] Germanguz I., Lev D., Waisman T., Kim C.-H., Gitelman I. (2007). Fourtwistgenes in Zebrafish, Four Expression Patterns. Dev. Dyn..

[16] Yang D.-C., Tsai C.-C., Liao Y.-F., Fu H.-C., Tsay H.-J., Huang T.-F., Chen Y.-H., Hung S.-C. (2011). Twist Controls Skeletal Development and Dorsoventral Patterning by Regulating Runx2 in Zebrafish. PLoS ONE.

[17] Knopf F., Hammond C.L., Chekuru A., Kurth T., Hans S., Weber C.W., Mahatma G., Fisher S., Brand M., Schulte-Merker S. (2011). Bone Regenerates via Dedifferentiation of Osteoblasts in the Zebrafish Fin. Dev. Cell.

[18] Sinha K.M., Zhou X. (2013). Genetic and Molecular Control of Osterix in Skeletal Formation. J. Cell. Biochem..

[19] Lawrence E.A., Kague E., Aggleton J.A., Harniman R.L., Roddy K.A., Hammond C.L. (2018). The Mechanical Impact of col11a2 Loss on Joints; Col11a2 Mutant Zebrafish Show Changes to Joint Development and Function, Which Leads to Early-Onset Osteoarthritis. Philos. Trans. R. Soc. B.

[20] Niu P., Zhong Z., Wang M., Huang G., Xu S., Hou Y., Yan Y., Wang H. (2017). Zinc finger transcription factor Sp7/Osterix acts on bone formation and regulates col10a1a expression in zebrafish. Sci. Bull..

[21] Hu Z., Chen B., Zhao Q. (2019). Hedgehog Signaling Regulates Osteoblast Differentiation in Zebrafish Larvae through Modulation of Autophagy. Boil. Open.

[22] Aceto J., Nourizadeh-Lillabadi R., Marée R., Dardenne N., Jeanray N., Wehenkel L., Aleström P., Van Loon J.J.W.A., Muller M. (2015). Zebrafish Bone and General Physiology Are Differently Affected by Hormones or Changes in Gravity. PLoS ONE.

[23] Mitchell R., Huitema L., Skinner R., Brunt L., Severn C., Schulte-Merker S., Hammond C.L. (2013). New Tools for Studying Osteoarthritis Genetics in Zebrafish. Osteoarthr. Cartil..

[24] Bergen D.J.M., Kague E., Hammond C.L. (2019). Zebrafish as an Emerging Model for Osteoporosis: A Primary Testing Platform for Screening New Osteo-Active Compounds. Front. Endocrinol..

[25] Flores M.V.C., Ni Lam E.Y., Crosier K.E., Crosier P.S. (2008). Osteogenic Transcription Factor Runx2 Is a Maternal Determinant of Dorsoventral Patterning in Zebrafish. Nature.

[26] Chen Z., Song Z., Yang J., Huang J., Jiang H. (2019). Sp7/osterix positively regulates dlx2b and bglap to affect tooth development and bone mineralization in zebrafish larvae. J. Biosci..

[27] Felber K., Elks P.M., Lecca M., Roehl H.H. (2015). Expression of Osterix Is Regulated by FGF and Wnt/β-Catenin Signalling During Osteoblast Differentiation. PLoS ONE.

[28] Hadzhiev Y., Lele Z., Schindler S., Wilson S.W., Ahlberg P.E., Strähle U., Müller F. (2007). Hedgehog Signaling Patterns the Outgrowth of Unpaired Skeletal Appendages in Zebrafish. Bmc Dev. Boil..

[29] Rotllant J., Liu D., Yan Y.-L., Postlethwait J.H., Westerfield M., Du S.-J. (2008). Sparc (Osteonectin) Functions in Morphogenesis of the Pharyngeal Skeleton and Inner Ear. Matrix Boil..

[30] Gistelinck C., Gioia R., Gagliardi A., Tonelli F., Marchese L., Bianchi L., Landi C., Bini L., Huysseune A., Witten P.E. (2016). Zebrafish Collagen Type I: Molecular and Biochemical Characterization of the Major Structural Protein in Bone and Skin. Sci. Rep..

[31] Yokokura T., Kamei H., Shibano T., Yamanaka D., Sawada-Yamaguchi R., Hakuno F., Takahashi S.-I., Shimizu T. (2017). The Short-Stature Homeobox-Containing Gene (shox/SHOX) Is Required for the Regulation of Cell Proliferation and Bone Differentiation in Zebrafish Embryo and Human Mesenchymal Stem Cells. Front. Endocrinol..

[32] Sawada R., Kamei H., Hakuno F., Takahashi S.-I., Shimizu T. (2014). In Vivo Loss of Function Study Reveals Theshort Stature Homeobox-containing(shox) Gene Plays Indispensable Roles in Early Embryonic Growth and Bone Formation in Zebrafish. Dev. Dyn..

[33] Zhang D., Wang J., Zhou C., Xiao W. (2017). Zebrafish Akt2 Is Essential for Survival, Growth, Bone Development, and Glucose Homeostasis. Mech. Dev..

[34] Ito-Amano M., Nakamura Y., Morisaki M., He X., Hayashi M., Watanapokasin R., Kato H. (2014). Temporal and Spatial Expression Patterns of Bone Morphogenetic Protein 3 in Developing Zebrafish. Open Rheumatol. J..

[35] Stewart S., Gomez A.W., Armstrong B.E., Henner A., Stankunas K. (2014). Sequential and Opposing Activities of Wnt and BMP Coordinate Zebrafish Bone Regeneration. Cell Rep..

[36] Asharani P., Keupp K., Semler O., Wang W., Li Y., Thiele H., Yigit G., Pohl E., Becker J., Frommolt P. (2012). Attenuated BMP1 Function Compromises Osteogenesis, Leading to Bone Fragility in Humans and Zebrafish. Am. J. Hum. Genet..

[37] Kondo M. (2007). Bone Morphogenetic Proteins in the Early Development of Zebrafish. Febs J..

[38] Gebuijs I.G.E., Raterman S.T., Metz J.R., Swanenberg L., Zethof J., Bos R.V.D., Carels C.E.L., Wagener F.A.D.T.G., Hoff J.V.D. (2019). Fgf8a Mutation Affects Craniofacial Development and Skeletal Gene Expression in Zebrafish Larvae. Boil. Open.

[39] Huitema L.F.A., Apschner A., Logister I., Spoorendonk K.M., Bussmann J., Hammond C.L., Schulte-Merker S. (2012). Entpd5 Is Essential for Skeletal Mineralization and Regulates Phosphate Homeostasis in Zebrafish. Proc. Natl. Acad. Sci. USA.

[40] Neacsu C.D., Grosch M., Tejada M., Winterpacht A., Paulsson M., Wagener R., Tagariello A. (2011). Ucmaa (Grp-2) Is Required for Zebrafish Skeletal Development. Evidence for a Functional Role of Its Glutamate γ-Carboxylation. Matrix Boil..

[41] Brandão A.S., Brito A.B., Lourenço R., Borbinha J., Soares A.R., Mateus R., Jacinto A. (2019). Yap Induces Osteoblast Differentiation by Modulating Bmp Signalling During Zebrafish Caudal Fin Regeneration. J. Cell Sci..

[42] Edsall S.C., A Franz-Odendaal T. (2010). A Quick Whole-Mount Staining Protocol for Bone Deposition and Resorption. Zebrafish.

[43] Witten P.E., Hansen A., Hall B.K. (2001). Features of Mono- and Multinucleated Bone Resorbing Cells of the ZebrafishDanio Rerio and Their Contribution to Skeletal Development, Remodeling, and Growth. J. Morphol..

[44] Cao L., Moriishi T., Miyazaki T., Iimura T., Hamagaki M., Nakane A., Tamamura Y., Komori T., Yamaguchi A. (2011). Comparative Morphology of the Osteocyte Lacunocanalicular System in Various Vertebrates. J. Bone Miner. Metab..

[45] Suniaga S., Rolvien T., Scheidt A.V., Fiedler I.A.K., Bale H.A., Huysseune A., Witten P.E., Amling M., Busse B. (2018). Increased Mechanical Loading through Controlled Swimming Exercise Induces Bone Formation and Mineralization in Adult Zebrafish. Sci. Rep..

[46] Mackay E.W., Apschner A., Schulte-Merker S. (2013). A Bone to Pick with Zebrafish. Bonekey Rep..

[47] Fiaz A.W., Léon-Kloosterziel K.M., Gort G., Schulte-Merker S., Van Leeuwen J.L., Kranenbarg S. (2012). Swim-Training Changes the Spatio-Temporal Dynamics of Skeletogenesis in Zebrafish Larvae (Danio Rerio). PLoS ONE.

[48] Doherty A.H., Ghalambor C.K., Donahue S.W. (2015). Evolutionary Physiology of Bone: Bone Metabolism in Changing Environments. Physiology..

[49] Nakagawa N., Kinosaki M., Yamaguchi K., Shima N., Yasuda H., Yano K., Morinaga T., Higashio K. (1998). RANK Is the Essential Signaling Receptor for Osteoclast Differentiation Factor in Osteoclastogenesis. Biochem. Biophys. Res. Commun..

[50] Lacey D., Timms E., Tan H.-L., Kelley M., Dunstan C.R., Burgess T., Elliott R., Colombero A., Elliott G., Scully S. (1998). Osteoprotegerin Ligand Is a Cytokine That Regulates Osteoclast Differentiation and Activation. Cell.

[51] Kitamura K.-I., Takahira K., Inari M., Satoh Y., Hayakawa K., Tabuchi Y., Ogai K., Nishiuchi T., Kondo T., Mikuni-Takagaki Y. (2013). Zebrafish Scales Respond Differently to in Vitro Dynamic and Static Acceleration: Analysis of Interaction Between Osteoblasts and Osteoclasts. Comp. Biochem. Physiol. Part. A Mol. Integr. Physiol..

[52] Negishi-Koga T., Shinohara M., Komatsu N., Bito H., Kodama T., Friedel R.H., Takayanagi H. (2011). Suppression of Bone Formation by Osteoclastic Expression of Semaphorin 4D. Nat. Med..

[53] Carbonare L.D., Valenti M.T., Zanatta M., Donatelli L., Cascio V.L. (2009). Circulating Mesenchymal Stem Cells with Abnormal Osteogenic Differentiation in Patients With Osteoporosis. Arthritis Rheum..

[54] Valenti M.T., Carbonare L.D., Mottes M. (2016). Osteogenic Differentiation in Healthy and Pathological Conditions. Int. J. Mol. Sci..

[55] De Franceschi L., Gabbiani D., Giusti A., Forni G.L., Stefanoni F., Pinto V.M., Sartori G., Balocco M., Zotto C.D., Valenti M.T. (2020). Development of Algorithm for Clinical Management of Sickle Cell Bone Disease: Evidence for a Role of Vertebral Fractures in Patient Follow-up. J. Clin. Med..

[56] Sehring I.M., Weidinger G. (2019). Recent Advancements in Understanding Fin Regeneration in Zebrafish. Wiley Interdiscip. Rev. Dev. Boil..

[57] Mishra R., Sehring I., Cederlund M., Mulaw M., Weidinger G. (2020). NF-κB Signaling Negatively Regulates Osteoblast Dedifferentiation During Zebrafish Bone Regeneration. Dev. Cell.

[58] Ando K., Shibata E., Hans S., Brand M., Kawakami A. (2017). Osteoblast Production by Reserved Progenitor Cells in Zebrafish Bone Regeneration and Maintenance. Dev. Cell.

[59] Paul S., Schindler S., Giovannone D., Terrazzani A.D.M., Mariani F.V., Crump J.G. (2016). Ihha Induces Hybrid Cartilage-Bone Cells During Zebrafish Jawbone Regeneration. Development.

[60] Kegelman C.D., Mason D.E., Dawahare J.H., Horan D.J., Vigil G.D., Howard S., Robling A.G., Bellido T.M., Boerckel J.D. (2018). Skeletal Cell YAP and TAZ Combinatorially Promote Bone Development. Faseb J..

[61] Seo E., Basu-Roy U., Gunaratne P.H., Coarfa C., Lim D.-S., Basilico C., Mansukhani A. (2013). SOX2 Regulates YAP1 to Maintain Stemness and Determine Cell Fate in the Osteo-Adipo Lineage. Cell Rep..

[62] Halder G., Dupont S., Piccolo S. (2012). Transduction of Mechanical and Cytoskeletal Cues by YAP and TAZ. Nat. Rev. Mol. Cell Boil..

[63] Pan J.-X., Xiong L., Zhao K., Zeng P., Wang B., Tang F.-L., Sun N., Guo H.-H., Yang X., Cui S. (2018). YAP Promotes Osteogenesis and Suppresses Adipogenic Differentiation by Regulating β-Catenin Signaling. Bone Res..

[64] Xiong J., Almeida M., O’Brien C.A. (2018). The YAP/TAZ Transcriptional Co-Activators Have Opposing Effects at Different Stages of Osteoblast Differentiation. Bone.

[65] Fisher S., Jagadeeswaran P., Halpern M.E. (2003). Radiographic Analysis of Zebrafish Skeletal Defects. Dev. Boil..

[66] Fiedler I.A., Schmidt F.N., Wölfel E.M., Plumeyer C., Milovanovic P., Gioia R., Tonelli F., Bale H.A., Jähn K., Besio R. (2018). Severely Impaired Bone Material Quality in Chihuahua Zebrafish Resembles Classical Dominant Human Osteogenesis Imperfecta. J. Bone Miner. Res..

[67] Garbes L., Kim K., Ries A., Hoyer-Kuhn H., Beleggia F., Bevot A., Kim M.J., Huh Y.H., Kweon H.-S., Savarirayan R. (2015). Mutations in SEC24D, Encoding a Component of the COPII Machinery, Cause a Syndromic Form of Osteogenesis Imperfecta. Expand. Spectr. Baf-Relat. Disord..

[68] Zancan I., Bellesso S., Costa R., Salvalaio M., Stroppiano M., Hammond C., Argenton F., Filocamo M., Moro E. (2014). Glucocerebrosidase Deficiency in Zebrafish Affects Primary Bone Ossification through Increased Oxidative Stress and Reduced Wnt/β-Catenin Signaling. Hum. Mol. Genet..

[69] Artola M., Kuo C.-L., Lelieveld L.T., Rowland R., Van Der Marel G.A., Codée J.D.C., Boot R.G., Davies G.J., Aerts J.M.F.G., Overkleeft H.S. (2019). Functionalized Cyclophellitols Are Selective Glucocerebrosidase Inhibitors and Induce a Bona Fide Neuropathic Gaucher Model in Zebrafish. J. Am. Chem. Soc..

[70] Gregson C.L., Bergen D.J.M., Leo P., Sessions R.B., Wheeler L., Hartley A., Youlten S., Croucher P.I., McInerney-Leo A.M., Fraser W. (2019). A Rare Mutation in SMAD9 Associated with High Bone Mass Identifies the SMAD-Dependent BMP Signaling Pathway as a Potential Anabolic Target for Osteoporosis. J. Bone Miner. Res..

[71] Luo Q., Liu S., Xie L., Yu Y., Zhou L., Feng Y., Cai D. (2019). Resveratrol ameliorates glucocorticoid-induced bone damage in a zebrafish model. Front. Pharmacol..

[72] Huang H.-X., Lin H., Lan F., Wu Y.-F., Yang Z.-G., Zhang J. (2018). Application of Bone Transgenic Zebrafish in Anti-Osteoporosis Chemical Screening. Anim. Model. Exp. Med..

[73] Chen J.-R., Lai Y.-H., Tsai J.-J., Hsiao C.-D. (2017). Live Fluorescent Staining Platform for Drug-Screening and Mechanism-Analysis in Zebrafish for Bone Mineralization. Mol..

[74] Van Der Kraan P. (2013). Relevance of Zebrafish as an OA Research Model. Osteoarthr. Cartil..

[75] Van Gennip J.L.M., Boswell C.W., Ciruna B. (2018). Neuroinflammatory Signals Drive Spinal Curve Formation in Zebrafish Models of Idiopathic Scoliosis. Sci. Adv..

